# Familial Chylomicronemia Syndrome-Induced Acute Necrotizing Pancreatitis during Pregnancy

**DOI:** 10.1055/s-0040-1722173

**Published:** 2021-02-18

**Authors:** Julia Cristina Coronado Arroyo, Marcio José Concepción Zavaleta, Eilhart Jorge García Villasante, Mikaela Kcomt Lam, Luis Alberto Concepción Urteaga, Francisca Elena Zavaleta Gutiérrez

**Affiliations:** 1 Department of Obstetrics and Gynecology, Hospital Nacional Edgardo Rebagliati Martins, Lima, Peru; 2 Department of Endocrinology, Hospital Nacional Guillermo Almenara Irigoyen, Lima, Peru; 3 Department of Endocrinology, Hospital Nacional Daniel Alcides Carrion, Lima, Peru; 4 Department of Medicine, Universidad Privada Antenor Orrego, Trujillo, Peru; 5 Department of Pulmonology, Hospital Regional Docente de Trujillo, Universidad Nacional de Trujillo, Trujillo, Peru; 6 Department of Neonatology, Hospital Belen de Trujillo, Universidad Privada Antenor Orrego, Trujillo, Peru

**Keywords:** familial chylomicronemia, necrotizing pancreatitis, pregnancy

## Abstract

Acute pancreatitis is a rare condition in pregnancy, associated with a high mortality rate. Hypertriglyceridemia represents its second most common cause. We present the case of a 38-year-old woman in the 24 ^th^ week of gestation with a history of hypertriglyceridemia and recurrent episodes of pancreatitis. She was admitted to our hospital with acute pancreatitis due to severe hypertriglyceridemia. She was stabilized and treated with fibrates. Despite her favorable clinical course, she developed a second episode of acute pancreatitis complicated by multi-organ dysfunction and pancreatic necrosis, requiring a necrosectomy. The pregnancy was ended by cesarean section, after which three plasmapheresis sessions were performed. She is currently asymptomatic with stable triglyceride levels. Acute pancreatitis due to hypertriglyceridemia represents a diagnostic and therapeutic challenge in pregnant women, associated with serious maternal and fetal complications. When primary hypertriglyceridemia is suspected, such as familial chylomicronemia syndrome, the most important objective is preventing the onset of pancreatitis.

## Introduction

Acute pancreatitis is an uncommon complication of pregnancy, with an incidence of 1 case per between 1,000 and 10,000 pregnancies. 1 2 The most common causes of acute pancreatitis are gallstones and hypertriglyceridemia. The latter may be due to diabetes, obesity, pregnancy, diet, hypothyroidism, alcohol, sepsis, renal failure, and drugs, while genetic causes represent < 5% of cases, among which is familial chylomicronemia syndrome. 3

Multiple treatment modalities have been established that range from conservative management based on diet, exercise, and fibrates to other therapeutic options such as plasmapheresis, insulin, and heparin. 4

Acute pancreatitis during pregnancy can lead to maternal complications such as pancreatic necrosis, pancreatic abscess, and multi-organ failure as well as fetal complications such as prematurity, fetal distress, and death. 5 Therefore, timely diagnosis and treatment are important to reduce maternal-fetal morbidity and mortality.

We present the case of a pregnant woman who developed acute necrotizing pancreatitis secondary to familial chylomicronemia syndrome.

## Case Description

A 38-year-old female patient, secundigravida, in the 24 ^th^ week of gestation, presented to the emergency room with somnolence and oppressive epigastric pain radiating to the back, which was associated with bilious emesis. She was diagnosed with hypertriglyceridemia at the age of 20 years old and was treated sporadically with 600 mg gemfibrozil TID. She also had a history of four episodes of pancreatitis, the first of which occurred at the age of 28 years old, and the last during the 16 ^th^ week of her current pregnancy; three of these episodes were due to hypertriglyceridemia, as her triglyceride levels were as high as 7,500 mg/dL, while the 4 ^th^ episode was due to biliary etiology, for which she underwent laparoscopic cholecystectomy. No record of alcoholism or contributory family history was found.

**Fig. 1:**
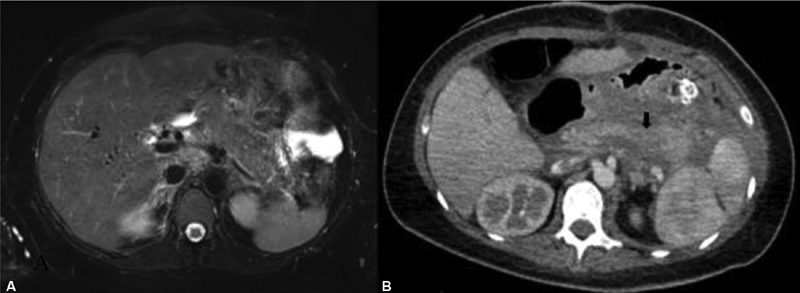
( **A** ) Abdominal T2-weighted magnetic resonance, performed during pregnancy, shows an edematous and heterogeneous pancreas, with surrounding edema. ( **B** ) Abdominal computed tomography scan with contrast, performed in the postpartum, shows area of hypoperfusion where the pancreatic body and head meet, suggestive of an area of necrosis.

Upon admission, general examination revealed weight: 60 Kg, height: 158 cm, temperature of 37.2°C, blood pressure of 70/40 mm Hg, heart rate of 136 beats/minute and respiratory rate of 28 breaths/minute. Relevant findings in the physical examination were marked pallor, decreased passage of vesicular murmur at the base of the lungs, and pain in the epigastrium without peritoneal signs. Laboratory tests revealed lipemic serum with the following results: leukocyte count, 7,200 cells/mm3 (neutrophils, 70%; band cells, 15%); hemoglobin, 13.3 g/dL; platelet count, 161,000 cells/mm3; serum glucose, 160 mg/dL; serum creatinine, 1 mg/dL; ALT, 126 U/L; AST, 100 U/L; amylase, 365 U/L; lipase, 305 U/L; C-reactive protein, 20.5 mg/L; albumin, 3.2 g/dL; sodium, 152 mEq/L; potassium, 4.6 mEq/L; corrected serum calcium, 6.2 mg/dL; HDL cholesterol, 34 mg/dL; LDL cholesterol, 28 mg/dL; triglycerides, 1,130 mg/dL; and apolipoprotein B (apoB) 48.7 mg/dL (reference value, 55–125 mg/dL). An abdominal ultrasound revealed decreased pancreatic echogenicity and a heterogeneous lesion 9 × 7 cm in size in the tail of the pancreas, which was complemented by magnetic resonance imaging (MRI), in which signs of acute pancreatitis were observed ( Fig. 1A ).

**Fig. 2:**
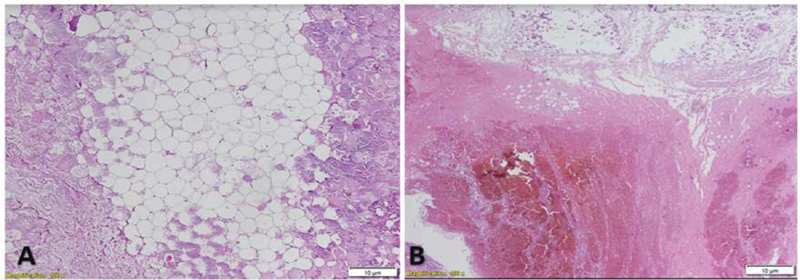
Histological changes of acute necrotizing pancreatitis with hematoxylin-eosin staining. ( **A** ) Lipid vacuoles immersed in pancreatic tissue with necrotic zones in the periphery. ( **B** ) Necrotic pancreatic tissue, where hemorrhagic focus is evident.

**Fig. 3:**
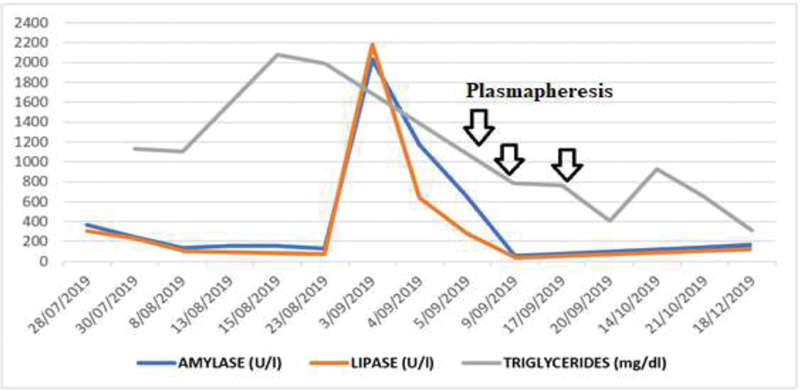
Serum amylase, lipase and triglyceride levels. Notice the elevation of pancreatic enzymes, compatible with the second episode of acute pancreatitis, which required necrosectomies. Likewise, a fall in triglyceride levels after plasmapheresis sessions is evidenced.

The initial treatment included fluid replacement, vasopressors, antibiotic therapy, and analgesia. Afterward, the patient was transferred to the intensive care unit (ICU) with the diagnosis of septic shock due to acute pancreatitis (APACHE II score, 8 points) secondary to severe hypertriglyceridemia. Upon hemodynamic stabilization and improvement in mental status, 160 mg fenofibrate QID and parenteral nutrition were initiated. The patient exhibited clinical improvement until day 39 of hospitalization, at which point she developed intense abdominal pain, hemodynamic instability, fetal bradycardia, and elevation of pancreatic enzymes. An emergency cesarean section was performed, which resulted in the delivery of a severely depressed male with a birth weight of 914 g. He was then transferred to the neonatal ICU, where he died on day 7 of life due to necrotizing enterocolitis. The neonate's triglyceride levels were 1,000 mg/dL. During surgery, 900 mm ^3^ of pus was found, which led to identification of the pancreas as the origin. During the postpartum period, the patient's clinical condition deteriorated, and she developed lung injury that required invasive mechanical ventilation, liver and kidney dysfunction, and bacteremia due to *Pseudomonas aeruginosa.* Hepatosplenomegaly and pancreatic necrosis were identified by abdominal computed tomography (CT) scan with contrast ( Fig. 1B ); as a result, the patient underwent two necrosectomy procedures, after which a histological analysis confirmed the lipid etiology ( Fig. 2 ). In terms of managing refractory hypertriglyceridemia, three plasmapheresis sessions were required, which resulted in favorable clinical and laboratory outcomes ( Fig. 3 ), which allowed to continue treatment outside the ICU during the remainder of her hospitalization, with subsequent medical discharge.

Currently, the patient is asymptomatic, is being treated with 160 mg fenofibrate BID and 25,000 IU pancreatic enzymes QID, has adopted a fat-restricted diet and consumes foods high in omega-3 fatty acids. Her fasting triglyceride levels range from 170 to 250 mg/dL and remains euglycemic.

## Discussion

Acute pancreatitis during pregnancy is a rare condition with high maternal-fetal mortality. 5 6 7 The most frequent cause is biliary, while the second most frequent cause is hypertriglyceridemia, which is associated with between 4 and 14.4% of all cases. 8 9 The etiology also varies according to the trimester of gestation 5 ; the cause in the first trimester is usually biliary, whereas, in the second and third trimesters, the cause is hypertriglyceridemia. A relationship has been reported between severity and etiology, considering that the cause is biliary in 39% of mild cases and that 58% of moderate and 100% of severe cases are due to hypertriglyceridemia. 5 Our patient experienced three episodes of pancreatitis during the second trimester, and the etiology of all episodes, the last of which was severe, was hypertriglyceridemia.

The higher incidence of hypertriglyceridemia during pregnancy is due to an increase in estrogens, progestogens, and human placental lactogen, which reduce the activity of lipoprotein lipase (LPL) by 85%. 10 Likewise, estrogens increase the hepatic synthesis of triglycerides and VLDL. 11 In contrast, insulin resistance increases, which lowers LPL activity in adipocytes. 12 In general, the concentration of triglycerides typically increases 2 to 3 times, 5 8 especially in the third trimester. However, the triglyceride concentration rarely exceeds 300 mg/dL, 11 except in patients with defects in lipid metabolism who develop severe hypertriglyceridemia, 11 such as our patient. Two theories explain why hypertriglyceridemia causes acute pancreatitis. One theory proposes that high levels of chylomicrons increase the viscosity of plasma, inducing ischemia in the pancreatic capillaries, which in turn generates acidosis and activates trypsinogen. 7 13 14 According to the other theory, the increase in triglyceride metabolism leads to increased production of free fatty acids, which causes cytotoxic damage to pancreatic acinar cells. 8

Hypertriglyceridemia can cause acute pancreatitis when the triglyceride levels are > 1,000 mg/dL or if they are between 500 and 999 mg/dL and associated with lipemic serum. 6 It can be primary or secondary to diabetes, obesity, pregnancy, and alcoholism, among other conditions. 14 15 16 Familial chylomicronemia syndrome is an autosomal recessive disorder caused by a mutation in the *LPL* gene and is characterized by severe hypertriglyceridemia and a poor response to traditional lipid-lowering agents, which causes recurrent episodes of acute pancreatitis. 14 17 In our case, the early age at onset of symptoms, triglyceride levels > 10 mmol/L (885 mg/dL) in 3 consecutive blood samples, a triglyceride/total cholesterol ratio > 5, reduced levels of apoB, and decreased levels of HDL and LDL cholesterol suggest familial chylomicronemia syndrome as the etiology. 17 18 A genetic analysis to identify specific mutations was not performed given that this exam is not available in Peru.

The diagnosis of acute pancreatitis during pregnancy requires two of the following three criteria: clinical, laboratory, and imaging findings. The symptoms do not differ from other presentations of acute pancreatitis, but peritoneal signs may be absent because stretching of the anterior abdominal wall leads to distancing from the area of inflammation; moreover, the size of the uterus limits the movement of the omentum toward the inflamed area. 8 Our patient presented upper abdominal pain and vomiting, but no evidence of peritonism. Regarding the etiology, identifying the signs of hypertriglyceridemia, such as lipemic serum, xanthomas, lipemia retinalis, and hepatosplenomegaly is important. 10 11 Our patient presented lipemic serum and hepatosplenomegaly. In terms of laboratory findings, the levels of amylase and lipase typically increase more than three times their normal values. However, in 50% of cases, amylase levels may be normal or low due to the presence of a serum amylase inhibitor. 8 Our patient had fluctuations in amylase and lipase levels during hospitalization, which were correlated with the clinical evolution. In terms of imaging, ultrasound is the preferred modality since it is safe and confirms biliary etiology. 8 Magnetic resonance imaging is indicated if ultrasound is unsuccessful. 8 In our case, both abdominal ultrasound and abdominal MRI without contrast revealed signs of acute pancreatitis.

Initial treatment includes fasting, hydration and analgesia. Then, measures such as a low-fat diet should be implemented to decrease triglyceride intake. 8 When enteral nutrition is impossible, intravenous lipids should be considered only when the triglyceride level is < 250 mg/dL. 8 Fibrates increase clearance, which decreases the triglyceride concentration by 50%. 13 16 The use of plasmapheresis is reserved for refractory cases, lactic acidosis, organic dysfunction and in cases in which the triglyceride levels exceed 1,000 mg/dL. 6 11 These are lowered by 70% after each session. 8 16 Furthermore, heparin and insulin increase the action of LPL, 8 16 and because insulin accelerates the breakdown of chylomicrons, it serves as an alternative when plasmapheresis is contraindicated and when the serum glucose is > 500 mg/dL. 11 In our case, the hypertriglyceridemia was refractory to fenofibrate therapy and dietary restrictions, and required three sessions of plasmapheresis and cesarean section. In relation to labor, it produces a rapid fall in estrogen levels; thus, triglyceride levels are also decreased. 8 The pregnancy should be terminated in 24 to 48 hours if clinical deterioration occurs, 6 7 as was the case with our patient.

Maternal mortality ranges from 20 to 37%, while fetal mortality ranges from 50 to 60%, which are related to the severity of the pancreatitis. 4 5 11 15 The primary factors that influence the prognosis are early diagnosis and disease management. 7 For this reason, when primary hypertriglyceridemia is suspected, preventing the onset of pancreatitis is crucial, 13 since severe complications, such as pancreatic necrosis and shock, can develop in addition to maternal and perinatal pathologies, such as preeclampsia, diabetes, macrosomia, prematurity, and stillbirth. 10 11 In the reported case, the complications developed in the mother were pancreatic necrosis and lung, liver, and kidney dysfunction, while the newborn was born prematurely and died.

Once acute pancreatitis has resolved, it is recommended that the lipid profile and physical activity be strictly monitored and that nutritional therapy based on a low-fat diet with a high content of omega-3 fatty acids be consumed. 11 Fibrates can be effective in patients with residual LPL activity. 18 The goal of therapy should be reducing triglyceride levels below the threshold for significant chylomicronemia (750 to 880 mg/dL) to reduce the risk of pancreatitis and improve the quality of life. 19

## Conclusion

Acute pancreatitis due to hypertriglyceridemia represents a diagnostic and therapeutic challenge in pregnancy, as it is associated with serious maternal and fetal complications. Therefore, considering primary causes in cases of severe hypertriglyceridemia is important. Finally, additional studies are needed to gather clinical data to establish guidelines for the management of acute pancreatitis secondary to hypertriglyceridemia in pregnancy.
